# Cannabidiol Regulates PPARγ-Dependent Vesicle Formation as well as Cell Death in A549 Human Lung Cancer Cells

**DOI:** 10.3390/ph15070836

**Published:** 2022-07-06

**Authors:** Yoon-Jong Park, Han-Heom Na, In-Seo Kwon, Yu-Na Hwang, Hye-Jin Park, Tae-Hyung Kwon, Jin-Sung Park, Keun-Cheol Kim

**Affiliations:** 1 Department of Biological Sciences, College of Natural Sciences, Kangwon National University, Chuncheon 24341, Korea; dbswhd1598@kangwon.ac.kr (Y.-J.P.); hanhum01@kangwon.ac.kr (H.-H.N.); kwoin087@kangwon.ac.kr (I.-S.K.); qha1259@kangwon.ac.kr (Y.-N.H.); hotliver4642@kangwon.ac.kr (H.-J.P.); 2 Kangwon Center for System Imaging, Kangwon National University, Chuncheon 24341, Korea; 3 Department of Research and Development, Chuncheon Bioindustry Foundation, Chuncheon 24341, Korea; taehyung0218@naver.com; 4 Korean Pharmacopuncture Institute, Seoul 07525, Korea; omd9330@hotmail.com

**Keywords:** cannabidiol (CBD), PPARγ, clathrin, adaptin, vesicle formation

## Abstract

Extracts of phytocannabinoids from *Cannabis sativa* have been studied for therapeutic purposes. Although nonpsychoactive CBD has been studied as a promising anticancer drug because it induces apoptosis in many cancer cells, it is also known to induce several physiological changes. In this study, we clarify the functional role it plays in the morphological characteristics of intracellular vesicle formation as well as apoptosis in A549 human lung cancer cells. CBD treatment shows growth inhibition at concentrations above 20 μM, but FACS analysis shows low efficacy in terms of cell death. Microscopic observations suggest that multiple vesicles were detected in the cytoplasmic region of CBD-treated A549 cells. CBD treatment upregulates apoptosis-related proteins, such as p53, PARP, RIP1, RIP3, Atg12, and Beclin, indicating that CBD regulates several types of cell death. CBD treatment also induced E-cadherin, PPARγ, clathrin, β-adaptin, and Tsg101, also known to be cellular-differentiation inducers or vesicle-formation components. Treatment combining CBD with GW9662, a PPARγ inhibitor, reduced CBD-induced cytoplasmic vesicle formation. This indicates that PPARγ regulates the vesicle-formation mechanism. However, CBD-treated E-cad KO clones did not show this regulatory mechanism. These results elucidate the pharmacological and molecular networks associated with CBD in PPARγ-dependent vesicle formation and the induction of apoptosis.

## 1. Introduction

Phytocannabinoids, which can be extracted from hemp plants, have been used for therapeutic purposes [1,2,3]. ∆9-tetrahydrocannabinol (THC) and cannabidiol (CBD) have been metabolically synthesized from cannabigerol (CBG) via the acid form (THCA or CBDA) with the catalytic action of THCA synthase (EC 1.21.3.7) or CBDA synthase (EC 1.21.3.8) [4]. Whereas THC has been known to offer potential therapeutic benefits for pain relief, nausea control, and appetite stimulation, as well as anti-inflammatory effects, its applications in many countries have been limited by law because of its psychoactive properties [5]. In contrast, the US FDA has approved nonpsychoactive CBD for treating epilepsy, as well as for application in treating other disorders, such as inflammatory diseases, chronic neurodegenerative diseases, and cancers [6].

CBD shows robust antiproliferative and proapoptotic effects in many types of cancer cells and inhibits migration, invasion, and metastasis [7,8,9]. CBD reportedly activates CB receptors in cancer cells, induces cell cycle arrest through the ERK pathway, and reduces cell proliferation, while inhibiting the PI3K/AKT pathway [10]. The TRPV1 receptor’s mediating activity also promotes ROS production to induce ER stress and upregulates TRIB3 to induce apoptosis [11]. CBD treatment increases the interaction with XIAP by promoting the release of the Smac proteins from mitochondria to the cytosol in gastric cancer cells [12]. It has also been reported that CBD exhibits anticancer effects by regulating PD-L1 expression in PC or PSC cells, suggesting that CBD suppresses KRAS-activated pathways by targeting PAK1 [13].

Other studies suggest that CBD’s cytotoxic effects might depend on the nature of phytocannabinoids, which can interact with the endocannabinoid system [14]. This so-called “entourage effect” could induce synergistic interactions among phytocannabinoids, and this effect could explain various pharmacokinetic properties, including substrate specificity, half-life, and regulatory functions with respect to target molecules [15]. CBDA concentrations were higher following cannabis extract administration than when administered as a single molecule in a mouse study, indicating that the crude extract provides a natural vehicle to substantially enhance CBDA concentrations [16].

Although phytocannabinoids exhibit anti-inflammatory and antitumorigenic properties through CB receptors, they also act through other receptors that can contribute to cancer progression, such as GPR55 [17]. Low concentrations of CBD may alter mitochondrial osmosis in BV2 glial cells, whereas high concentrations of CBD are known to induce damage by impairing mitochondrial complex activity in isolated mitochondria [18,19]. However, other studies have reported conflicting results at the same dosage range, with CBD improving mitochondrial health in both isolated mitochondria and mouse cardiac tissue and increasing cardiac tissue cell viability [20,21]. Therefore, it is possible that CBD may mediate different pharmacological activities depending upon cell types and concentration [22,23].

This study observed physiological changes in terms of cell death and intracellular vesicle formation following CBD treatment of A549 human lung cancer cells. Vesicle formation seems to have a regulatory effect on the growth and death of cancer cells. Although numerous papers have reported on CBD-induced cell death, it is still necessary to describe novel physiological functions and plausible regulatory networks.

## 2. Results

### 2.1. CBD Treatment Shows Growth Inhibition and Morphological Changes in A549 Cells

**Figure 1:**
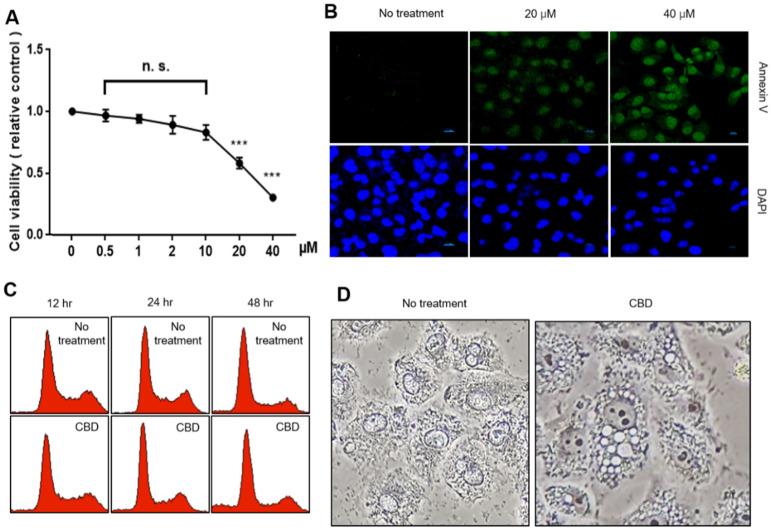
Growth inhibition and morphological changes due to CBD. (**A**) Cell viability was measured by MTT assay with CBD at various concentrations in A549 cells. Data are presented as mean ± SD. n.s.—no significance, *** *p* < 0.01 (Student’s *t*-test). (**B**) Dead cells were assessed with annexin V staining. (**C**) FACS analysis was performed with 20 μM of CBD-treated A549 cells at the indicated time points. (**D**) Cellular morphology was observed with a phase contrast microscope after 20 μM CBD for 48 h.

We performed an MTT assay to analyze the cell proliferation effects after treating the A549 cells with CBD (Figure 1A). We did not observe growth inhibition in the CBD range of less than 10 μM, but it was distinct in the range of 20 μM or more. To examine whether growth inhibition is related to cell death, we performed an annexin V staining experiment, which is known to show an early marker protein during cell death induction. We observed annexin V-positive cells after treatment with 20 μM CBD or more (Figure 1B). Annexin V is commonly used to detect various stages of apoptotic cells due to its ability to bind to phosphatidylserine, a marker of apoptosis localized in the plasma membrane. We also analyzed cell cycle distributions to examine the ratios between living and dead cells. CBD treatment increased the number of dead cells by a small proportion during a 48 h treatment (Figure 1C). However, most cells maintained their cell cycle distribution under CBD treatment. Cell morphology dramatically changed during CBD treatment (Figure 1D). Aggregated cells were observed when treated with CBD, and numerous vesicles appeared in the cytoplasmic region. The vesicles did not take an Oil Red O solution stain, indicating that multiple vesicle formations may not be associated with adipocyte-like differentiation (Supplementary Figure S1). Cytoplasmic-accumulated black spots were also observed via electron microscopy (Supplementary Figure S2). Thus, CBD treatment inhibits cell growth and alters cellular characterization.

### 2.2. CBD Upregulates Multiple Proteins for Cell Death or Differentiation

**Figure 2:**
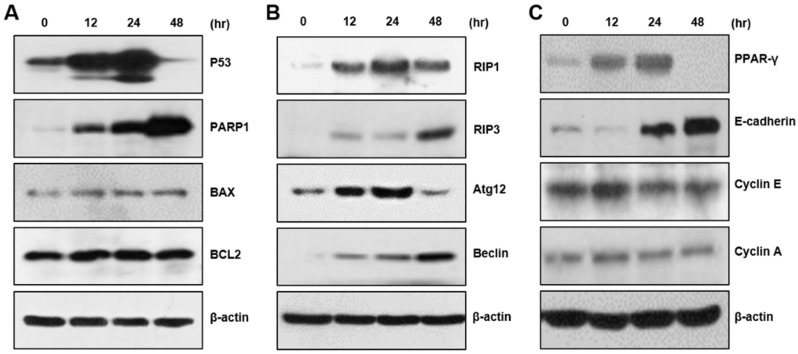
Upregulation of multiple proteins by CBD treatment. (**A**) A Western blot analysis was performed on apoptosis-related proteins. P53 and PARP-1 were upregulated by CBD treatment, whereas Bax and BCL2 levels were not changed. (**B**) Necroptotic or autophagy marker proteins were increased by CBD treatment. (**C**) Levels of the differentiation-related proteins PPARγ and E-cadherin were increased by CBD treatment.

We examined the expression of cell death-related proteins. CBD increased the expression of p53 protein and PARP1, which are typical apoptosis protein markers. In addition, we observed cleaved PARP1 after 24 h of CBD treatment. However, we could observe no changes in BAX or BCL2 protein expression (Figure 2A). We also examined RIP1, RIP3, Atg12, and Beclin, which are marker proteins for necroptosis, pyroptosis, and autophagy, and observed that CBD treatment also increases the levels of these proteins (Figure 2B). To examine whether CBD regulates cellular differentiation, we performed a Western blot analysis for protein differentiation and adhesion and cell cycle progression. CBD upregulated PPARγ and E-cadherin proteins, although there were no distinct changes in cyclin protein levels (Figure 2C). These results suggest that CBD regulates the expression of many proteins involved in apoptosis, differentiation, and metastasis.

### 2.3. CBD Upregulates Protein Components of Vesicle Formation

**Figure 3:**
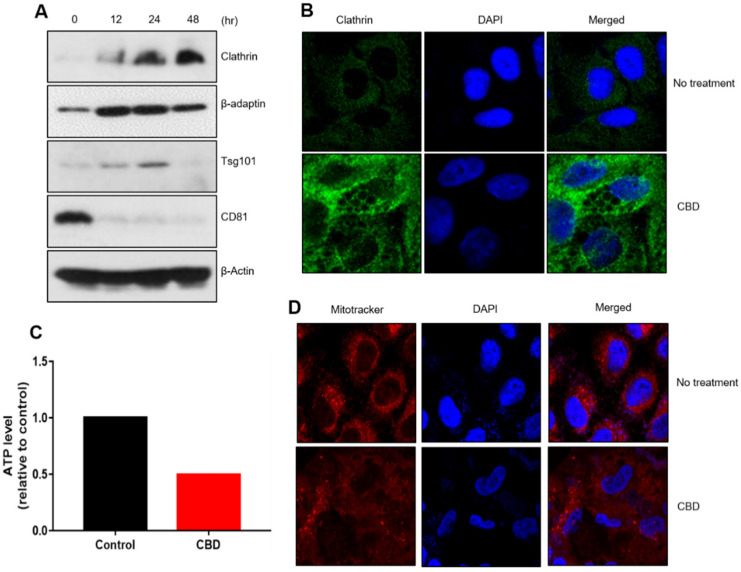
Regulation of vesicle-formation components and energy production by CBD. (**A**) Clathrin-coated components were regulated in CBD-treated A549 cells. Clathrin, ꞵ-adaptin, and Tsg101 protein levels were increased by CBD treatment, but CD81 expression was downregulated by CBD. (**B**) Clathrin expression was also analyzed with an immunostaining experiment. (**C**) Intracellular ATP levels were determined after 20 μM CBD for 24 h. (**D**) A549 cells were treated with 20 μM CBD for 24 h and subjected to MitoTracker staining.

To analyze whether CBD induces multiple vesicles in the cytoplamic region, we examined expressional changes for vesicle-formation proteins. Western blot analysis showed that the expressions of clathrin, β-adaptin, and Tsg101 were upregulated by CBD treatment, but that of CD81 was downregulated (Figure 3A). Immunostaining experiments revealed that increased clathrin protein was mainly detected around vesicles in the cytoplasmic region (Figure 3B). We investigated whether changes in cellular characteristics were associated with changes in metabolic activity. We measured adenosine triphosphate (ATP) production and glucose levels after CBD treatment. CBD treatment decreased intracellular ATP and glucose levels, suggesting that CBD altered cellular metabolic activity in A549 cells (Figure 3C, Supplementary Figure S3). MitoTracker staining analysis showed that although mitochondria in the control cells were localized around the nucleus of untreated A549 cells, CBD treatment dispersed this localization throughout the cytoplasm (Figure 3D). These results imply that cytoplasmic vesicle formation might be associated with changes in metabolic activity.

### 2.4. PPARγ Regulates Vesicle Formation in Relation to CBD

**Figure 4:**
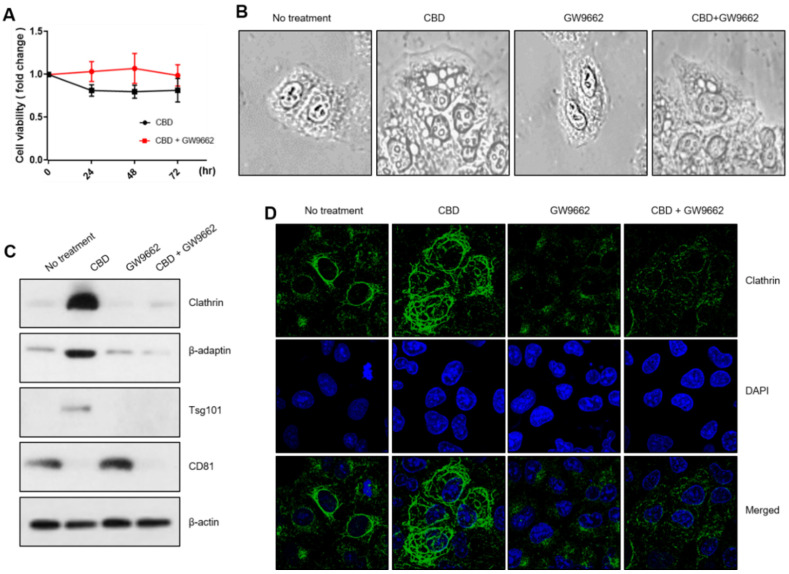
Regulation of vesicle-formation components by PPARγ. (**A**) MTT assay was performed after combinatory treatment with CBD and GW9662, and PPARγ inhibitor. (**B**) Morphological changes were observed with a microscope after combinatory treatment with CBD and GW9662. (**C**) A Western blot analysis was performed to determine whether vesicle-formation components are regulated with inhibition of PPARγ. (**D**) Clathrin expression was also analyzed with an immunostaining experiment after combinatory treatment with CBD and GW9662.

**Figure 5:**
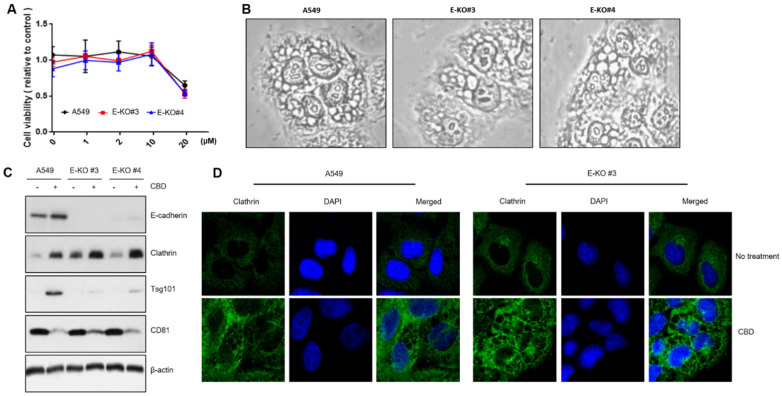
Irrelevance of E-cadherin to vesicle-formation components. (**A**) An MTT assay was performed with E-cadherin KO clones. (**B**) Morphological changes were microscopically observed with CBD-treated E-cadherin KO clones. (**C**) A Western blot analysis was performed to determine whether vesicle formation is regulated in E-cadherin KO clones. (**D**) Clathrin expression was also analyzed with an immunostaining experiment in A549 cells and E-cadherin KO clones.

We used GW9662, a PPARγ inhibitor, and CBD to analyze the molecular networks involved in vesicle-formation changes. The cell growth rate was slightly increased with a combined CBD and GW9662 treatment (Figure 4A). Microscopic analysis showed that GW9662 treatment was associated with no distinct vesicle-formation patterns. However, the combined treatment of GW9662 with CBD lessened CBD-induced cytoplasmic vesicle formation, suggesting that PPARγ regulates the mechanism for its formation (Figure 4B). Western blot analysis showed that the increased expression of such vesicle-formation-related proteins as clathrin, β-adaptin, and Tsg101 was also dramatically decreased by coadministration with GW9662 (Figure 4C). Similarly, an immunostaining experiment showed that clathrin was increased in the cytoplasmic region, but this was reversed by cotreatment with GW9662, indicating that vesicle formation might be closely associated with PPARγ activity (Figure 4D). We also investigated a plausible interaction between vesicle formation regulation and E-cadherin expression. We compared CBD vesicle formation between A549 cells and E-cadherin knockout clones prepared with CRISPR/cas9. Vesicle formation and cell growth did not differ significantly between A549 cells and E-cadherin KO clones (Figure 5A,B). Additionally, Western blot and immunostaining experiments showed no difference in vesicle-formation-related proteins, indicating that E-cadherin expression does not depend on vesicle formation (Figure 5C,D).

## 3. Discussion

CBD, a nonpsychoactive substance, not only shows potential pharmacological effects in neurological diseases, but is also receiving growing attention as a potential anticancer agent [24,25]. It has also been suggested that CBD has a greater antitumor effect than psychoactive THC at a similar concentration [26,27]. Generally, CBD inhibits cell proliferation in different cancer cell lines, including colorectal, breast, prostate, and head and neck squamous cancer cell lines [28,29]. However, although CBD has shown positive anticancer properties in in vitro cell-based experiments and in in vivo xenograft tumor models, clinical attempts to treat cancer patients have been few. Therefore, the pharmacological effects of CBD have yet to be fully explored at the molecular level.

Depending on cell type, the optimal CBD concentration range for antitumor effects varies greatly from 0.01 to 100 mM [30]. In this study, we investigated the antitumor effects that 20 µM of CBD has on A549 cells, based on our observation that an MTT assay shows that 20 µM of CBD inhibits A549 lung cancer cell growth. CBD concentrations in this range also increase the expression of annexin V, which is known to be an early-stage apoptosis marker. This indicates that CBD treatment promotes cell death [31]. FACS analysis suggests that CBD can induce cell death in a small population of A549 cells without disrupting the cell cycle in most other cells. We noted multiple intracellular cytoplasmic vesicles in viable cells with microscopic observations, which indicated that CBD can induce apoptosis depending upon the concentration. CBD has been known to have excellent anticancer effects on in vitro cancer cells, but these effects might be associated with the cell types or treatment concentrations [32]. High concentrations of CBD treatment may possibly show a greater inhibition of A549 cell growth [33]. However, CBD can also induce morphological changes in many types of cancer cells [34,35]. CBD-caused vesicle formation has also been seen in in vitro experimentation and has been suggested to be a mechanism of cell death via autophagy in MCF7 cells [36]. It has been reported that dying cells relocate or degrade organelles, including the endoplasmic reticulum, Golgi apparatus, and lysosomes [37]. Although our current mitoTracker analysis showed delocalization of mitochondria, the possibility of functional changes to various organelles still remained. We therefore suggest that CBD has various pharmacological effects in terms of morphological and functional changes, such as vesicle formation and cell death.

CBD induces apoptotic cell death by increasing the expression of p53 and PARP. CBD also increases the expression of RIP1, RIP3, Atg12, and Beclin, which suggests that it regulates pyroptosis and autophagy. Given that the dead cell population induced by 20 μM CBD represented only a fraction of the total cell population, the expression of these proteins may be a necessary but insufficient condition for several types of cell death. Moreover, since CBD also regulates E-cadherin and PPARγ, cell death is likely not a unique process induced by CBD but one which can be paralleled by such concurrent processes as EMT transition and metabolic regulation.

Our current study suggests that CBD also induces the expression of clathrin, β-adaptin, and Tsg101 proteins, which have been described in cytoplasmic vesicle formation [38]. We investigated the correlation between the expressional changes in E-cadherin or PPARγ and multiple intracellular vesicles, as seen in CBD-treated A549 cells. The PPARγ antagonist GW9662 counteracted the CBD antiproliferative effects, suggesting that CBD-induced vesicle formation may be associated with cell growth inhibition as well as such decreased metabolic capacities as ATP synthesis and glucose uptake. However, the E-cadherin KO clones exhibited no distinct changes in growth inhibition or vesicle formation, indicating that E-cadherin does not affect intracellular vesicle formation, but that it does affect cell–cell interactions which we probably did not detect in this study.

As a lipophilic molecule, CBD is a chemical compound with high cell membrane permeability [39]. Thus, CBD modulates intracellular target proteins to induce various cellular changes depending upon CBD-binding receptors or CBD concentration in the context of cells and microenvironments [40,41]. Similarly, CBD may be synergistic with combinatory therapies using such anticancer drugs as doxorubicin or cisplatin [42,43]. CBD has been suggested to possibly reduce drug resistance by long-term clinical treatment with anticancer drugs [44,45].

Thus, we suggest that CBD has multiple pharmacological effects, including intracellular vesicle formation and death of cancer cells. We also reckoned that a physiologically active CBD concentration is an important determinant in cell death or morphological changes. Therefore, these results will be very useful for future pharmacological CBD applications once the overall CBD target gene regulatory network has been investigated.

## 4. Materials and Methods

### 4.1. Cell Culture and Reagents

We maintained A549 human lung cancer cells in RPMI-1640 supplemented with 10% fetal bovine serum and 1% penicillin–streptomycin and cultured them at 37 °C in a CO_2_ incubator (Welgene Inc., Seoul, Korea). We observed the cellular morphology with a phase contrast microscope or a scanning electron microscope (SEM) (CX-200TM, COXEM, Daejeon, Korea). CBD was purchased from the Cayman company, USA, and dissolved in DMSO; aliquots were stored at −20 °C.

### 4.2. Cell Viability Assay and Annexin V Staining

Cell viability was measured using an MTT (dimethylthiazole-2′,5′-diphenyl-2-H-tetrazolium bromide) assay. The MTT was diluted in PBS (phosphate-buffered saline). We seeded 1 × 10⁴ cells in 96-well plates for the MTT assay. The next day, each well was treated with CBD in triplicate and incubated until the indicated time points. For the MTT assay, we incubated the plate in 1 X MTT solution at 37 °C for four hours. Formazan crystals were dissolved in MTT solution by replacing 200 μL of dimethylsulfoxide. Absorbance was measured at a wavelength of 570 nm using a microplate reader (Bio-Rad, Hercules, CA, USA). Cell death was analyzed using annexin V-FITC (BioVision, Milpitas, CA, USA). The 549 cells were treated with 20 μM CBD for 24 h. The cells were incubated with 5 μL of annexin V for 10 min in the dark at room temperature. Immunofluorescent cells were observed with a confocal microscope.

### 4.3. FACS Analysis

The A549 cells were cultured after treatment with CBD. The cells were harvested and washed twice in PBS and fixed in 75% ethanol for 24 h. Cells were stained with propidium iodide (50 mg/mL) for 30 min. Cell cycle distribution was analyzed via flow cytometric analysis according to Becton Dickinson’s protocols.

### 4.4. Western Blot Analysis

Proteins were extracted using RIPA lysis buffer (25 mM Tris-HCl pH 7.6, 150 mM NaCl, 1% NP-40, 1% sodium deoxycholate, 0.1% SDS) supplemented with proteasome inhibitors. Protein concentrations were measured using Bradford reagent (Thermo Fisher, Waltham, MA, USA). We separated 50 μg of protein with SDS-PAGE gel electrophoresis and transferred this onto a polyvinylidene fluoride (PVDF) membrane. The PVDF membrane was blocked using 5% skimmed milk for one hour at room temperature, followed by incubation with specific primary antibodies. Afterwards, the membrane was washed and incubated with appropriate secondary antibodies at room temperature for one hour. Finally, the proteins were detected using an ECL protein detection kit (GE healthcare, Chicago, IL, USA). Primary antibodies for caspase 9, phospho-RIP1, RIP1, RIP3, GSDMD, and IL-1β were purchased from CST (Danvers, MA, USA). We obtained the PARP, Bax, Bcl2, cyclin D3, cyclin A, cyclin B, and β-actin from Santa Cruz (Dallas, TX, USA).

### 4.5. Immunostaining or MitoTracker Staining

We cultured the A549 cells on coverslips, washed them with PBS, and fixed them with 100% methanol solution for 10 min. Cells were permeabilized with a solution of Triton X-100 dissolved in 0.2% PBS for 10 min. After blocking this for one hour using 5% skimmed milk in PBS, we incubated them with a primary antibody (1:100) at room temperature for two hours. The primary antibody was dissolved in 5% skimmed milk and used. Then, the cells were washed with PBS and incubated with secondary Alexa 488 goat antimouse IgG antibodies (1:500; Abcam, Boston, MA, USA). The secondary antibodies were diluted 1:200 in 5% skimmed milk and used. Next, the cells were stained with DAPI for counterstaining. The disruption of mitochondria function was assessed with a fluorescence-based assay using MitoTracker solution reagent (Thermo Fisher, Waltham, MA, USA). A549 cells were seeded onto a cover slip and treated with CBD. Afterwards, the medium was removed and treated with 100 nM of MitoTracker for 30 min. Coverslips were mounted on a glass slide with an anti-discoloration reagent. We analyzed fluorescence images with the Gangwon Convergence Bioimaging System Center’s confocal microscope (Ts2, Nikon, Tokyo, Japan).

### 4.6. Metabolic Analysis

We measured intracellular glucose levels with a glucose uptake kit (Promega, Madison, WI, USA). The A549 cells were seeded onto a 3 × 10^5^ inch 6-well plate. The 2-deoxyglucose (2-DG) detection reagents were prepared according to the manufacturer’s recommendation. After adding 2-DG to each well, we added stop and neutralization buffers. Luminescence was measured using a luminometer (Schroff, Straubenhardt, Germany). Cellular ATP level was also measured with an ATP Determination Kit reagent (Thermo Fisher, Waltham, MA, USA). The standard solution volume (10 mL) contains the following components: 0.5 mL 20X reaction buffer, 0.1 mL 100 mM dithiothreitol, 0.5 mL 10 mM D-luciferin, and 2.5 µL 5 mg/mL firefly recombinant. CBD-treated cell lysate was prepared using a 1X luciferase lysis buffer. Afterwards, the cell lysate was analyzed for bioluminescence activity using a luminometer.

## 5. Conclusions

Recently, the pharmacological efficacy of CBD has been focused on in many types of disease models. In this study, we have shown that CBD treatment upregulated cell death proteins, such as p53, PARP, RIP1, RIP3, Atg12, and Beclin. In addition, CBD treatment also induced E-cadherin, PPARγ, clathrin, β-adaptin, and Tsg101, also known to be cellular-differentiation inducers or vesicle-formation components. We have also shown that PPARγ regulates the vesicle-formation mechanism. Therefore, not only have we confirmed cell death induction by CBD, we have also confirmed the regulation of vesicle formation by PPARγ in various cancer models. Detailed molecuclar insights would enhance the therapeutic utilization of CBD without side effects. Cellular proliferation and death could be determined by a variety of molecular regulations in cellular networks composed of a variety of proteins. Moreover, the physiological changes in cells will be closely related to functional changes in intracellular organelles. We suggest that an understanding of the complex intracellular network system is necessary to optimize the pharmacological efficacy of CBD. These steady research efforts will make significant progress in increasing the utilization of CBD, which has been restricted by legal regulations in some countries.

## Data Availability

Data is contained within the article and Supplementary Material.

## Supplementary Materials

The following supporting information can be downloaded at: https://www.mdpi.com/article/10.3390/ph15070836/s1, Figure S1: Oil Red O staining after CBD treatment. Figure S2. SEM observation on cellular morphology. Figure S3. Measurement of glucose level after CBD treatment. Figure S4. Preparation E-cadherin KO clones with CRISPR/cas-9 system.
