# Supplementary material for: Cannabidiol Regulates PPARγ-Dependent Vesicle Formation as well as Cell Death in A549 Human Lung Cancer Cells
Source: Pharmaceuticals (Basel). 2022 Jul 6;15(7):836. doi: 10.3390/ph15070836 (PMC9319361; doi:10.3390/ph15070836)
Supplement: Supplementary file 1 [file pharmaceuticals-15-00836-s001.zip › pharmaceuticals-1783893-supplementary.pdf]

Supplementary Figure Legends.

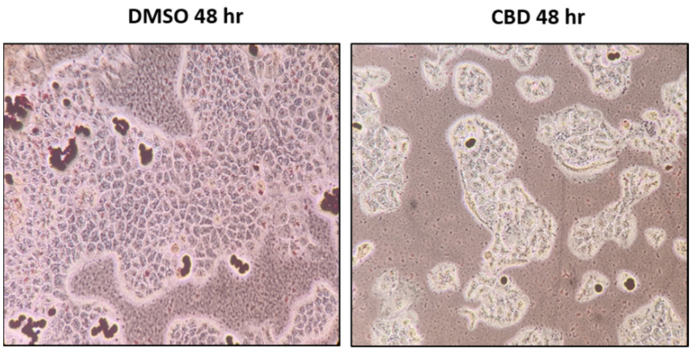

Figure S1. Adipocytic differentiation was measured with Oil Red O staining. Intracelllar vesicles formed by CBD treatment are not related to adipocyte differentiation.

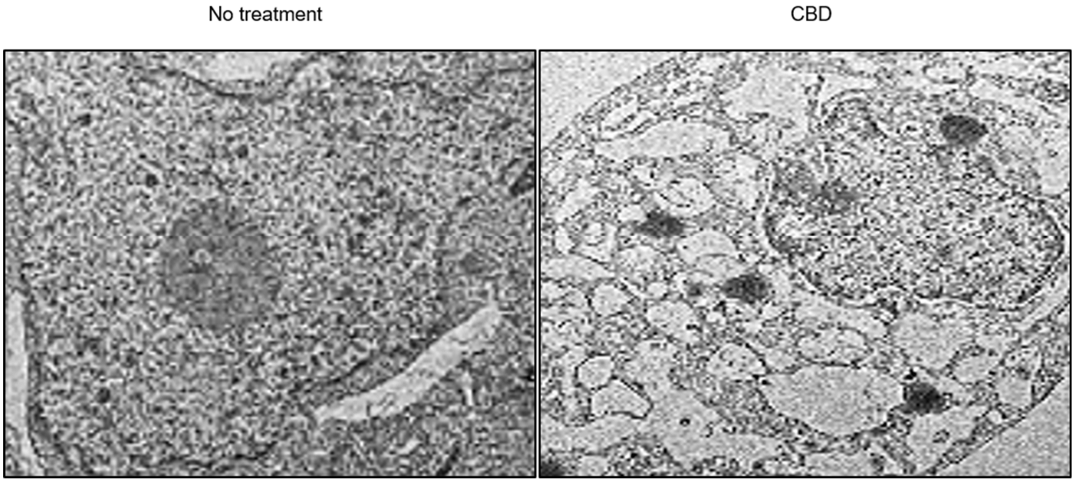

Figure S2. Cellular morphology was also observed with scanning electron microscopy (SEM) after 20 μM CBD for 48 hours.

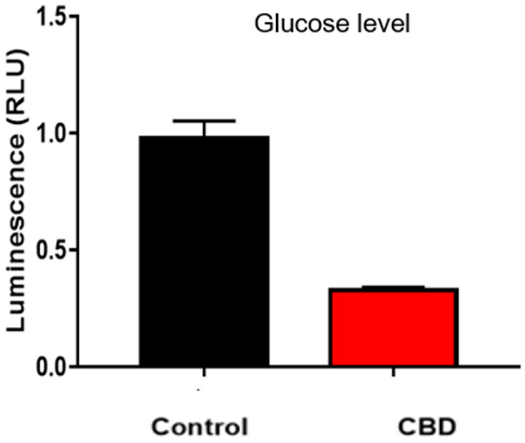

Figure S3. Intracelluar glucose level was determined after 20 μM CBD for 24 hours.

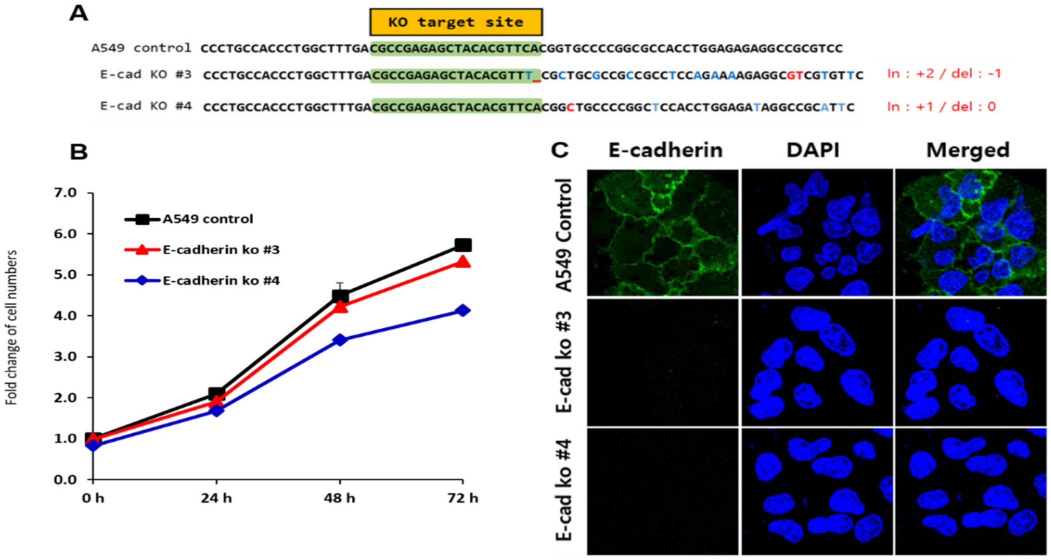

Figure S4. Preparation E-cadherin KO clones with CRISPR/cas-9 system. A. KO clones were established with RNA guidance for targeting exon2 of E-caherin genes. Sequencing data shows several mutations at the target sequences. B. The cell growth rates were slightly slowed in KO clones compared to A549 cells. C. E-cadherin expression was also assessed after CBD-treated condition in E-cadherin KO clones compared to A549 cells.
